# Assessing the Value of Omalizumab for Pediatric Asthma in China: A Multicriteria Decision Analysis

**DOI:** 10.3390/healthcare13192385

**Published:** 2025-09-23

**Authors:** Yuncui Yu, Wang Cao, Yue Xiao, Jing Wei, Huijie Huang, Ang Li, Mingyang Zhao, Lihua Hu, Chittawan Poonsiri, Yot Teerawattananon, Alec Morton, Peng Guo

**Affiliations:** 1 Clinical Research Center, Beijing Children’s Hospital, Capital Medical University, National Center for Children’s Health, Beijing 100045, China; yuyuncui7@126.com (Y.Y.); loiscw@163.com (W.C.); weijingbch@126.com (J.W.); 2 Department of Pharmacy, Beijing Children’s Hospital, Capital Medical University, National Center for Children’s Health, Beijing 100045, China; hlh97@sina.com; 3 National Health Development Research Center, National Health Commission, Beijing 100044, China; moonxy@126.com; 4 Department of Allergy, Beijing Children’s Hospital, Capital Medical University, National Center for Children’s Health, Beijing 100045, China; huangfu0523@126.com (H.H.); liang15656035332@163.com (A.L.); 5 Department of Epidemiology and Health Statistics, School of Public Health, Capital Medical University, Beijing 100069, China; 18910788991@163.com; 6 Health Intervention and Technology Assessment Program, Ministry of Public Health, Nonthaburi 11000, Thailand; chittawan.p@hitap.net (C.P.); Yot.t@hitap.net (Y.T.); 7 Saw Swee Hock School of Public Health, National University of Singapore, Singapore 119077, Singapore; alec.morton@strath.ac.uk; 8 Department of Management Science, Strathclyde Business School, University of Strathclyde, Glasgow G11XQ, UK

**Keywords:** medicine selection and prioritization, asthma, omalizumab, multicriteria decision analysis (MCDA)

## Abstract

**Background/Objectives**: This study aimed to apply a multicriteria decision analysis to assess the comprehensive value of omalizumab for moderate to severe pediatric asthma in China. **Methods**: A multidisciplinary panel of 17 experts assessed the value of omalizumab plus the standard of care (SOC) using SOC alone as a comparator. We developed a hierarchical criteria system with six main domains and 15 specific criteria. To establish a comprehensive evidence matrix, we integrated findings from a systematic literature review (SLR) and a real-world pharmacovigilance study based on the FAERS database. The overall estimated value of each strategy was obtained by combining the criterion weights with the score of each strategy in each criterion. A sensitivity analysis was conducted to validate the robustness of the results. **Results**: According to the AHP methods, the following weights were assigned to the criteria: safety (38.55%), effectiveness (28.85%), economics (9.65%), innovation (8.24%), accessibility (7.84%), and applicability (6.88%). Based on the evidence matrix, omalizumab plus SOC scored higher than the SOC in effectiveness (2.53 vs. 1.94) and innovation (0.70 vs. 0.15). When the weight and score of each strategy in each criterion were combined, the overall estimated values were 7.40 points for omalizumab plus SOC and 7.19 points for SOC. **Conclusions**: Adding omalizumab was assessed as a conditionally recommended strategy for treating moderate to severe asthma in Chinese children.

## 1. Introduction

Bronchial asthma (BA) is a complex disease characterized by chronic airway inflammation, afflicting over 300 million people worldwide and causing approximately 250,000 annual deaths [1]. In China, asthma accounted for approximately 1 million disability-adjusted life years (DALYs) across all ages in 2010, with children (aged 0–14 years) representing the majority of cases at a prevalence of 3%—a rate rising steadily annually [2,3]. Allergic asthma affects 60–80% of BA patients and involves the release of Th2 cytokines and the production of immunoglobulin E (IgE) antibodies [4]. While most patients can better control their asthma symptoms with standardized multidisciplinary triage and management, approximately 20% of patients with moderate-to-severe asthma do not respond effectively to standard-of-care (SOC) treatments such as high-intensity inhaled corticosteroids (ICS) and long-acting beta2 agonists (LABA) [5]. These patients often face an increased risk of asthma exacerbation, a significant decline in lung function, and higher rates of emergency department visits and hospitalizations, which are more than 15 times higher than those of mild-to-moderate patients [1].

One of the primary factors in the development and progression of allergic asthma is increased serum levels of total and specific IgE. Most pediatric patients with moderate-to-severe asthma have higher mean IgE levels than those with mild disease [6]. Omalizumab is a humanized anti-immunoglobulin E monoclonal antibody that interferes with the binding of Ig E to the receptor FcεRI on the surface of mast cells and eosinophils. This interference inhibits mast cell and eosinophil activation, decreases inflammatory mediators’ release, reduces inflammatory cell recruitment, and prevents airway tissue remodeling and functional alterations [7]. As the first targeted drug for asthma treatment, omalizumab has been approved for treating IgE-mediated moderate-to-severe allergic asthma in patients over the age of 6 years in the U.S in 2003. It was also approved for marketing in China for the treatment of IgE-mediated moderate to severe allergic asthma patients aged 6 years and older, as well as chronic spontaneous urticaria patients aged 12 years and older. It is recommended by the Global Initiative for Asthma (GINA) and China’s guidelines for the diagnosis and prevention of childhood bronchial asthma due to its ability to reduce acute exacerbations, decrease the risk of deterioration, and lower the required dose of ICS while being well tolerated [8,9]. However, the high cost of omalizumab treatment raises concerns about its economic value as an add-on therapy, which varies according to country [10,11].

Several prominent health technology assessment (HTA) agencies, such as the UK’s National Institute for Health and Care Excellence (NICE) and the Canadian Agency for Drugs and Technologies in Health (CADTH), have evaluated omalizumab [12,13]. Their reports indicate that omalizumab reduces the risk of asthma exacerbations in adult patients, while exhibiting a safety profile similar to placebo. A consistent limitation noted across these reports is the scarcity of evidence concerning pediatric use. Regarding cost-effectiveness, the assessments revealed considerable variation, attributable to divergent evaluations of asthma-related mortality, quality-of-life benefits, efficacy metrics, and patient characteristics [14]. Critically, these investigations failed to implement analysis using multi-criteria decision analysis (MCDA), while the dated evidence necessitates updating. Nowadays, China is advancing comprehensive drug evaluation, requiring both government and healthcare institutions to make decisions like formulary inclusion based on multidimensional assessments of drug value. Additionally, the comprehensive value of omalizumab needs evaluation based on clinical needs to support rational clinical selection and use. Consequently, leveraging recent evidence updates and policy shifts (e.g., NRDL inclusion), this study aim to employ MCDA methodology to comprehensively evaluate omalizumab’s clinical value, establishing an evidence-based foundation for rational medication use and medication policy optimization.

## 2. Materials and Methods

### 2.1. Study Design

**Figure 1:**
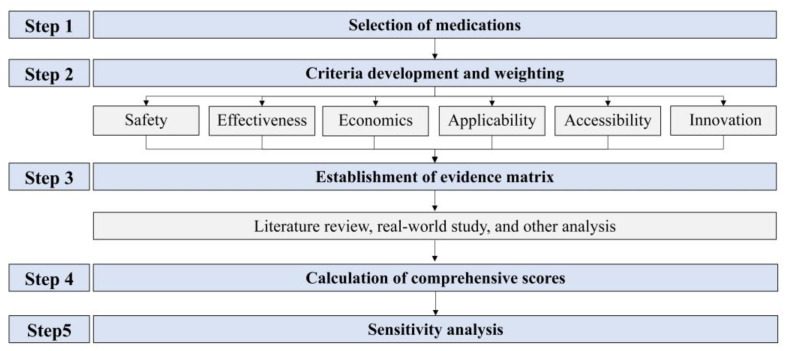
The flow chart for the comprehensive evaluation of pediatric medicines in clinical settings based on multicriteria decision analysis.

This study adhered to Chinese guidelines and the ISPOR MCDA Good Practice Guidelines Checklist [15]. The workflow consisted of five steps, as illustrated in Figure 1. In accordance with pediatric clinical practice, omalizumab plus SOC was designated as the study group, while SOC alone served as the control group.

### 2.2. Criteria Development and Weighting

To ensure a nationally representative and multidisciplinary experts panel, a structured multi-tiered selection process was employed: representative provinces and municipalities across China’s major administrative regions (e.g., Beijing, Tianjin, Shandong, Henan, Anhui, Jiangsu, Shanxi) were first selected; leading Grade A tertiary children’s hospitals within these regions were then identified; and senior clinicians, pharmacists specializing in allergy and respiratory medicine from these hospitals, as well as methodological experts from national HTA agencies and academic institutions, were invited. The final panel consisted of 17 experts with the following composition: seven pediatricians (including three allergists and four respiratory specialists), six hospital pharmacists (with expertise in allergy, respiratory medicine, medicine consultation, or medication management), one pharmacoepidemiologist, two health economists, and one pharmaceutical policy specialist—resulting in a balanced ratio of clinical, pharmacy, and methodological expertise (7:6:4). Prior to their participation, all experts were required to disclose any potential conflicts of interest. No major conflicts were reported that were deemed to influence the study’s objectivity.

Based on the guideline [16], we established a preliminary hierarchical criteria system for asthma medicines, with domains governing subordinate selection criteria. This framework was refined through expert panel discussions to eliminate duplication and ensure completeness, resulting in six finalized domains: effectiveness, safety, economics, applicability, accessibility, and innovation.

Criterion weights for the MCDA model—reflecting panelists’ priorities in treatment comparisons—were determined using the analytic hierarchy process (AHP) [17,18]. The methodology involved: (1) building the criteria hierarchy, (2) conducting pairwise comparisons using a 9-point scale for all criteria, and (3) calculating priority weights with Yaahp 10.3 software. Experts’ authority coefficients (Cr) were derived as the mean of their influence (Ca) and familiarity (Cs): Cr = (Ca + Cs)/2.

### 2.3. Establishment of Evidence Matrix

#### 2.3.1. Systematic Literature Review (SLR)

The information sources for this study included authoritative databases such as PubMed, EMBase, The Cochrane Library, China National Knowledge Infrastructure (CNKI), Wanfang Database, China Science and Technology Journal Database (VIP) and China Biomedical Literature Database, among others.

Literature was collected from standardized processes before August 2025 (Figure S1). The following criteria were established using the PICOS principle: (P) Children (0–18 years) with allergic asthma; (I) Omalizumab + SOC; (C) SOC; (O) Effectiveness (severe exacerbation rate, acute attack frequency, lung function improvement, Global Evaluation of Asthma Effectiveness (GETE) excellent response, children’s Asthma Control Test (C-ACT) score, pediatric Asthma Quality of Life Questionnaire (PAQLQ) score, absenteeism, corticosteroid use), safety (general/severe adverse drug reactions (ADR) or adverse drug events (ADE)), and economics (daily/course costs, cost-effectiveness); (S) clinical practice guidelines (CPGs), HTA reports, systematic reviews, RCTs, cohort studies, and economic studies. Exclusions included duplicates, inaccessible texts, and non-English/Chinese literature.

A standardized, pre-piloted data extraction form was developed to systematically collect relevant information from each included study. Data extraction was performed independently by two researchers to ensure accuracy and consistency. Any discrepancies between the two researchers were resolved through discussion or, when necessary, by consulting a third researcher All extracted data were cross-checked against the original studies for validation.

#### 2.3.2. Real World Study

The real-world study presented in this section constitutes a pharmacovigilance analysis aimed at signal detection, which employs the US FDA Adverse Event Reporting System (FAERS)—a database containing spontaneously reported adverse event data obtained from real-world clinical environments. The ADR reports associated with omalizumab use in children (0–18 years) were extracted from the FAERS database covering the period from January 2004 to November 2023. Data querying, extraction, cleaning, and standardization were performed using OpenVigil 2.1 (https://openvigil.sourceforge.net (accessed on 20 November 2023)). Disproportionality analysis was conducted using the proportional reporting ratio (PRR) and reporting odds ratio (ROR) methods. A signal was considered positive by the PRR method if PRR > 2 and χ^2^ ≥ 4. For the ROR method, a positive signal required N ≥ 3 and the lower limit of the 95% confidence interval (95% CI) > 1. A potential statistically significant association between the drug and an ADE was only considered if both methods yielded a positive signal.

#### 2.3.3. Other Analysis

To evaluate accessibility, we analyzed the formulary inclusion status of omalizumab across 60 healthcare institutions nationwide, examining its availability in children’s hospitals, maternal and child health hospitals, and general hospitals. Affordability was assessed by calculating the annual treatment cost as a percentage of per capita disposable income. Benchmarking utilized 2024 income data (national average: ¥41,314; urban: ¥54,188; rural: ¥23,119). Other evidence collected for this study included medicine instructions and official webpages such as U.S. Food and Drug Administration (FDA) and China’s National Medical Products Administration (NMPA).

### 2.4. Calculation of Comprehensive Scores

The 17 experts were invited to score each criterion based on the evidence matrix (ESc), using a scale of 1 to 10. The scores were classified into three levels: poor (1 to 3), moderate (4 to 7), and excellent (8 to 10). The comprehensive score (CS) of a criterion equals criteria weight (Wc) multiplied by ESc (Formula (1)). To determine the overall value of each strategy, all the weighted scores of the considered criteria were summed up (Formula (2)).

CS (criterion) = Wc × ESc(1)

(2) $$CS\left(Strategy\;A\right)=\sum_{allcriteria}Wc\times ESc$$

### 2.5. Sensitivity Analysis

To address potential subjectivity in expert-driven weight allocation, sensitivity analysis was performed by sequentially adjusting domain weights while preserving their hierarchical order. Six alternative weight-allocation scenarios representing distinct preference-elicitation approaches were tested. Overall treatment scores were calculated separately for each scenario, with resultant rankings compared against the baseline. Consistent ordering across all six scenarios confirmed methodological robustness.

## 3. Results

### 3.1. Criteria Assessment and Weighting

**Figure 2:**
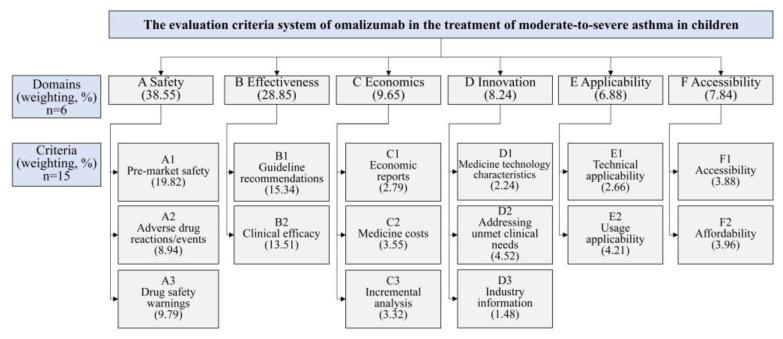
Criteria and weighting in the MCDA framework for evaluating omalizumab in treating moderate-to-severe allergic asthma in children.

The evaluation criterion system was built structured hierarchically, with six domains, including safety, effectiveness, economics, innovation, applicability, and accessibility and 15 overall criteria (Figure 2 and Table S1).

Seventeen experts participated in weight determination (Table S2). Cr analysis confirmed panel authority (all Cr > 0.8, Table S3). The results of the criteria weights revealed that the domain with the highest weight was safety (38.55%), followed by effectiveness (28.85%), economics (9.65%), innovation (8.24%), accessibility (7.84%), and applicability (6.88%), respectively. The three most important criteria were pre-market safety (19.82%), guideline recommendations (15.34%), and clinical efficacy (13.51%) (Figure 2).

### 3.2. Evidence Matrix

#### 3.2.1. Safety

Seven clinical guidelines affirm omalizumab’s favorable safety profile [19,20,21,22,23,24,25]. Five specify that treatment may induce systemic or local hypersensitivity reactions, typically mild-to-moderate and transient [20,21,23,24,25]. Two recommend administering injections exclusively in medical facilities equipped to manage anaphylaxis, with mandatory specialized training for personnel [19,22]. Supporting this, three HTA studies [12,13,14] and eight clinical trials [26,27,28,29,30,31,32,33] further confirmed omalizumab’s tolerability: Most ADRs were mild (e.g., abdominal pain, pyrexia, urticaria), with no treatment discontinuations or deaths attributable to AEs. Overall ADR incidence showed no statistical difference from placebo (Table 1).

FAERS database analysis identified 1678 pediatric ADR reports, with primary signals including urticaria, hypersensitivity reactions, respiratory distress, headache, and malaise (Table 2). Both FDA and NMPA have issued safety communications regarding potential cardiovascular/cerebrovascular event risks associated with omalizumab.

#### 3.2.2. Effectiveness

Eleven clinical guidelines affirm omalizumab’s effectiveness in asthma management, with seven specifically endorsing its use as add-on therapy for moderate-to-severe allergic asthma [19,20,21,22,23,24,25,36,37,38]. This consensus is supported by comprehensive evidence from three HTAs [12,13,14] and ten supplementary studies (three meta-analyses; seven observational studies) [26,27,28,29,30,31,32,33,34,35], which collectively demonstrate that omalizumab reduces risks of mild exacerbations and acute asthma attacks, decreases oral medication requirements (including OCS/ICS), lowers unplanned healthcare utilization and rescue medication dependence, and improves asthma control (Table 1).

#### 3.2.3. Economics

Four economic studies in pediatrics from four countries revealed divergent cost-effectiveness conclusions for omalizumab: two studies supported its cost-effectiveness, while two did not (Table 3). Results from the only available Chinese study indicated that add omalizumab therapy was not cost-effective versus standard therapy alone for severe allergic asthma in children [11].

The average price of omalizumab (150 mg/vial) was ¥1319.39. For an 8-year-old child (30 kg), a 12-month course cost 31,665.36 (¥86.75/day). As a Class B NRDL-listed drug, patient out-of-pocket cost reached ~¥400/vial in Beijing, reducing the annual course to ¥9600 (¥26.30/day) (Table 4).

#### 3.2.4. Applicability

Omalizumab is indicated for patients aged ≥ 6 years with allergic asthma or chronic spontaneous urticaria (CSU), featuring minimal age restrictions for pediatric use and safe excipient profiles. However, its subcutaneous administration (biweekly or monthly) may compromise adherence in children, while cold-chain storage requirements increase medication management costs.

#### 3.2.5. Accessibility

Our survey of formularies across 60 hospitals nationwide revealed an overall omalizumab coverage rate of 56.67% (34/60). Higher availability was observed in children’s specialty hospitals (12/20, 60%) and general hospitals (20/26, 60%), while maternal and child health hospitals showed lower coverage rate at 14.29% (Table 4).

From the disposable income perspective, annual omalizumab treatment costs for allergic asthma represented 22.30–52.28% of urban-rural household disposable income. This proportion decreased to 6.76–15.85% after reimbursement through national insurance (Table 4).

#### 3.2.6. Innovation

As the first anti-IgE targeted drug for asthma, omalizumab meets the medication needs of moderate to severe asthma patients whose symptoms cannot be effectively controlled by conventional treatments. Currently, there is no patent protection for it in China, with 1 original drug enterprise and 1 generic drug enterprise.

### 3.3. Overall Score Calculation

Following evidence matrix-based expert assessment, the omalizumab plus SOC group obtained higher mean scores than the SOC group in safety, effectiveness, and innovation dimensions based on the evidence matrix. However, the SOC group achieved higher scores in the dimensions of economics, applicability, and accessibility. Upon combining the weighting of each criterion with the scoring of each strategy, the omalizumab plus SOC group obtained an overall estimated value of 7.40 points, which was higher than the value obtained by the SOC group (7.19 points) (Table 5).

### 3.4. Sensitivity Analysis

Sensitivity analysis employing six distinct domain weight combinations (Table 6) demonstrated consistent strategy rankings across all scenarios. The omalizumab plus SOC group maintained primary positioning regardless of weighting variations, confirming model robustness to domain weight adjustments.

## 4. Discussion

To our knowledge, this is the first study to employ MCDA to evaluate the comprehensive value of omalizumab for children suffering from moderate to severe allergic asthma. Unlike previous studies focusing on isolated dimensions, our research systematically synthesizes multi-dimensional evidence to generate composite scoring and evidence-based recommendations. The findings of the study suggest that omalizumab remains relatively costly at present price and policies, and its addition to SOC treatment provides a conditionally recommended strategy.

In this study, a criterion system was established specifically for evaluating pediatric medicines. The system was constructed hierarchically, with six main domains at the top and 15 subordinate criteria below them. The weight of each criterion was determined using the AHP method, which is known for being reliable and user-friendly, and less prone to subjective bias compared to expert group discussions [17]. The results showed that medicine safety was the most important concern in pediatrics, followed by effectiveness, consistent with findings from our previous study [18]. The panelists ranked “pre-market safety” and “guideline recommendations” as the most important criteria, while “industry information” was considered the least important criterion. The experts gave similar attention to the remaining four domains, with slightly higher emphasis on economics and innovation. This ranking reflects the differing concerns between pediatrics and adults’ clinical settings.

The results showed that omalizumab plus SOC was significantly more effective and innovative than SOC treatments for asthma. Studies have shown that it reduces the chance of mild and acute exacerbations, decreases the need for oral medications such as OCS or ICS, lowers the number of unplanned clinic visits and emergency medication use, and improves overall asthma control [26]. This conclusion is supported by all experts and aligns with guideline recommendations [8,9]. In terms of safety, omalizumab plus SOC was essentially equivalent to SOC. The main difference is reflected in the drug safety warnings. Both the U.S. FDA and China’s NMPA have released information indicating that omalizumab may increase the risk of cardiovascular and cerebrovascular adverse events. However, based on pre-market and post-market evidence, ADEs to omalizumab were mostly mild, including abdominal pain, fever, and urticaria. The total incidence of ADRs was not significantly different from that of the placebo group [12].

It is important to note that omalizumab scored lower in most subcategories of economics. The cost-effectiveness of using omalizumab for treating allergic asthma in children varies greatly depending on the country, policy, and evaluation perspective [11,41]. In China, despite omalizumab being included in the national health insurance catalog, the drug price and incremental analyses show it does not provide economic advantages [11]. The high annual per capita treatment cost of omalizumab for children’s allergic asthma as a percentage of the annual per capita disposable income of urban and rural households, ranges from 24% to 60%. After medical insurance reimbursement, the proportion is around 7% to 18%. It is important to monitor any future adjustments to prices and changes in medical policies in the future.

In addition, omalizumab is slightly less applicable and accessible compared to SOC. While it has fewer age restrictions for pediatric use and its formulation excipients are safe according to the drug instructions, the medication is administered through subcutaneous injections, which may impact adherence in pediatric patients [42]. Furthermore, omalizumab requires refrigeration, potentially leading to higher drug management costs. Our data shows that the overall coverage rate of omalizumab in healthcare institutions is approximately 57%. Tertiary pediatric and general hospitals have a higher coverage rate of 60%, whereas maternity and child healthcare hospitals have a lower coverage rate of 14%. Although omalizumab has specific storage and management requirements, the survey results still indicate a need to enhance its availability in community or secondary hospitals.

The differences between the two strategies on various dimensions highlight the need to assess the comprehensive value from a holistic point of view. The results of the sensitivity analysis did not affect our final decision on medicine selection, indicating that the model is robust concerning different weights assigned to domains. Our study demonstrates that MCDA can provide a framework for integrating different sorts of evidence and the judgement from different experts at a local level. It should be noted that the results derived from this method serve only a reference for the decision-making process, which must also consider other factors such as local priorities and resources. Additionally, the study findings are subject to change should relevant policy adjustments occur or new evidence emerge.

The study is not exempt from some limitations. First, the assignment of weights and scores to different criteria was based on expert judgment, which can introduce bias. We employed the AHP methodology to minimize expert subjectivity. Second, further validation of the evaluation system’s scientific rigor, stability, and applicability is warranted. Third, as a context-specific MCDA, the generalizability of our findings to other countries or populations requires external validation. Fourth, due to the limited availability of evidence exclusively in pediatric populations, several studies including both pediatric and adult participants were incorporated. Finally, certain data were unavailable, such as Chinese real-world safety and effectiveness data and the costs of treating complications after taking omalizumab. Regular updates of evidence and results are necessary for this study in the future.

## 5. Conclusions

This MCDA study provides a structured and transparent evaluation of omalizumab’s value for pediatric moderate-to-severe asthma in China. Results indicate a modest advantage for omalizumab plus SOC over SOC alone (MCDA score: 7.40 vs. 7.19), supported by its effectiveness, innovation, and acceptable safety profile. However, economic concerns remain due to inconsistent cost-effectiveness outcomes—including a Chinese study reporting non-cost-effectiveness. Consequently, omalizumab may be conditionally recommended as an adjunct therapy within China, though the generalizability of these findings is limited by context-specific data and regional constraints. Future evaluations should prioritize updated real-world evidence on long-term safety, regional costs, and broader applicability.

## Figures and Tables

**Table 1:** Safety and effectiveness evidence summary from included literature.

ID	No. of Studies/Patients	Intervention	Control	Safety	Effectiveness
Lu Cheng, 2023 [26]	13 studies	OMZ	Placebo	AE of fever, injury, or trauma: OR = 1.91, 95%CI: 1.38, 2.64; No significant differences in all of the other AEs	Total IgE: ES = −1.22, 95%CI: 1.75, 0.70; FeNO: ES = −1.01, 95%CI: 1.55, 0.48; ACT and C-ACT: ES = 0.93, 95%CI: 0.65, 1.2; FEV1/FVC: ES = 0.32, 95%CI: 0.72,1.37
Dandan Lang, 2023 [27]	7 studies	ICS + OMZ	ICS + placebo	ADR: RR = 1.00, 95%CI: 0.98, 1.03; Serious ADR: RR = 0.53, 95%CI: 0.36, 0.77	Efficacy: RR = 1.24, 95%CI: 1.09, 1.41; Exacerbation within 24 weeks: RR = 0.55, 95%CI: 0.35, 0.85; Exacerbation within 52 weeks: RR = 0.52, 95%CI: 0.39, 0.71
Jean Bousquet, 2021 [34]	86 studies	Post-OMZ	Pre-OMZ	-	Good/excellent GETE at 16 weeks: 77%, RD = 0.77, 95% CI: 0.70, 0.84; Good/excellent GETE at 12 months: 82%, RD = 0.82, 95% CI: 0.73, 0.91; ACT decreased at 16 weeks (−1.14), 6 months (−1.56), and 12 months (−1.13); Severe exacerbations rate: RR = 0.41, 95%CI: 0.30; 0.56; The proportion of patients receiving OCS: RR = 0.59, 95% CI: 0.47; 0.75; Number of unscheduled physician visits: MD = −2.34, 95% CI: −3.54, −1.13
Xiang Li, 2023 [28]	200	Post-OMZ	Pre-OMZ	53 cases reported AEs: Mild AEs: 44 (83.0%); Moderate AEs: 9 (17.0%); Severe AEs: 0; Abdominal pain: 2 (1.0%); Fever: 2 (1.0%)	C-ACT score after 4 weeks: 18.90 ± 3.74 vs. 22.70 ± 3.70; Moderate to severe asthma exacerbations decreased after 4 to 6 months: 2.00 ± 5.68 episodes per person-year, *p* < 0.001; ICS daily dose: 0 (0–240 μg) vs. 160 (50–4000 μg), *p* < 0.001; PAQLQ: 154.90 ± 8.57 vs. 122.80 ± 27.15; FEV1%: 92.80 ± 10.50% vs. 89.70 ± 18.17%
Antonio Nieto García, 2020 [29]	484	Post-OMZ	Pre-OMZ	Cases with at least one AE: 21 (4.3%)	The annual number of MSE decreased: 7.9 ± 6.6 vs. 1.1 ± 2.0; Mild exacerbations: −64.4%, 95%CI: −71.1%, −57.7%; ICS daily dose: 867.3 ± 474.5 μg vs. 663.4 ± 431.4 μg
Noriko Nakamura, 2020 [30]	128	Post-OMZ	Pre-OMZ	ADR: 13 (10.2%), Pyrexia (2.4%), Urticaria (1.6%); AE: 60 (47.2%), SAE: 30 (23.6%)	Worsening of asthma symptoms requiring systemic steroid: 25.2% vs. 74.0%; Frequency of hospitalization: 54.0% vs. 85.0%. Visits to the emergency room: 43.6% vs. 78.2%; Absence from school: 36.4% vs. 78.2%
Hesham N. Tarraf, 2018 [31]	59	OMZ + SOC	SOC	No new safety signals	The proportion of patients receiving OCS: 81.1% at baseline vs. 52.8% at Week 16, *p* < 0.001; A daily dose of OCS decreased by 55% (*p*< 0.001); Exacerbations or missed days from work or school: n = 0, *p*< 0.001; ACQ-5 scores decreased: 3.23 vs. 1.75, *p* < 0.001
Weikun Chong, 2023 [32]	26	Post-OMZ	Pre-OMZ	Local and systemic adverse events were not reported; Liver and renal dysfunctions were not detected.	Increased C-ACT scores after 16 weeks: 15.57 ± 3.25 points vs. 24.98 ± 5.21 points, *p* < 0.001; Decreased FeNO: 31.55 ± 15.57 ppb vs. 19.86 ± 9.80 ppb, *p* = 0.0022; Decreased VAS scores: 6.40± 2.98 points vs. 0.85 ± 0.40 points, *p* < 0.001.
Norrice M. Liu, 2024 [35]	142	Post-OMZ	Pre-OMZ	-	64.1% had controlled according to GINA; In the last 12 months, 54.1% and 29.6% had at least one and two exacerbations, respectively; 7% were admitted to hospital due to exacerbation.
Lucia Caminiti, 2024 [33]	6	Post-OMZ	Pre-OMZ	Isolated episode of acute urticaria: 1 (16.6%); Injection site pain: 2 (33.3%); No severe AEs.	55% reduction from baseline in ACQ-5 scores after 16 weeks (*p* = 0.002); Only one patient (16.6%) achieved complete symptom control with a score < 1; No hospitalization and/or Emergency Departments admissions were observed due to asthma exacerbation.

OMZ: omalizumab; ADR: adverse drug reactions; AE: adverse events; SAE: Serious adverse events; ICS: inhaled corticosteroids; SOC: standard of care; IgE: immunoglobulin; ES: effect size; GETE: Global evaluation of treatment effectiveness; RD: risk difference; ACT: Asthma Control Test; OCS: oral corticosteroids; C-ACT: Children-Asthma Control Test; PAQLQ: Pediatric Asthma Quality of Life Questionnaire; MD: mean difference; MSE: moderate-to-severe exacerbations; FEV1/FVC: forced expiratory volume in 1 s/forced vital capacity; ACQ: Asthma Control Questionnaire; VAS: visual analogue scale; Mini Asthma Quality of Life Questionnaire.

**Table 3:** Economics evidence summary from included literature.

ID	Country	Outcomes	OMZ Cost-Effectiveness
Hideki Yoshikawa, 2016 [39]	Japan	Each OMZ-treated patient experienced an increase of 40.8 HFD in HFD every 2 years. The cost of each additional HFD was 20,868 yen	Yes
Hua Zhou, 2020 [11]	China	SOC plus OMZ required an additional expenditure of $49,047 per 0.232 QALY gained, with an ICER of $211,217 per QALY.	No
Carlos E. Rodriguez-Martinez, 2021 [40]	Colombia	The ICUR of OMZ compared to SOC was $82,748.1 per QALY.	No
María Nieto-Cid, 2023 [41]	Spain	The ICER per avoidance of moderate to severe exacerbations after 1 year was 2107 euros, increasing to 656 euros at 6 years of follow-up. At year 1 and year 6, for each 0.5-point improvement in ACQ-5, the ICER decreased from 2059 euros to 380 euros, and for each 3-point improvement in c-ACT, the ICER decreased from 3141 euros to 2322 euros.	Yes

OMZ: omalizumab; SOC: standard of care; HFD: hospital-free days; QALY: quality-adjusted life year; ICER: incremental cost-effectiveness ratio; ACQ-5: Asthma Control Questionnaire; c-ACT: Children-Asthma Control Test; ICUR: incremental cost-utility ratio.

**Table 4:** Analysis of economy and accessibility of omalizumab.

Category	Parameter	Pre-Reimbursement	Post-Reimbursement
Cost (¥ ^a^)	Drug price (/branch)	1319.39	400
	Daily average cost	86.75	26.3
	Course of treatment cost ^b^	31,665.36	9600
Medical institution equipping status (n, %)	Total, n = 60	34, 56.67%	-
	Pediatric hospitals, n = 20	12, 60.00%	-
	General hospitals, n = 26	20, 76.92%	-
	Maternal and child health hospitals, n = 14	2, 14.29%	-
Proportion of annual treatment costs in annual disposable income of resident households (%)	All residents	29.25	8.87
	Urban residents	22.30	6.76
	Rural residents	52.28	15.85

^a^ The exchange rate between the Chinese yuan (¥) and the US dollar ($) is about 7.17 (¥7.17 = $1). ^b^ The total treatment cost was calculated based on a 12-month regimen for an 8-year-old child (30 kg body weight), receiving 300 mg (2 vials) of omalizumab subcutaneously every 4 weeks.

**Table 5:** The overall score of omalizumab plus SOC versus SOC.

Domains	Criteria	ESc (Ten-Point Scale)		CS	
		OMZ + SOC	SOC	OMZ + SOC	SOC
Safety	A1 Pre-Market safety	7.71 ± 1.02	7.24 ± 1.96	1.53	1.43
	A2 Adverse drug reactions/events	7.71 ± 1.52	7.00 ± 2.22	0.69	0.63
	A3 Drug safety warnings	6.06 ± 1.95	7.82 ± 1.50	0.59	0.77
	Total	-	-	2.81	2.83
Effectiveness	B1 Guideline recommendations	8.06 ± 1.68	7.19 ± 1.18	1.16	1.04
	B2 Clinical efficacy	8.76 ± 0.64	6.71 ± 1.67	1.18	0.91
	Total	-	-	2.35	1.94
Economics	C1 Economic reports	7.24 ± 1.11	7.00 ± 1.33	0.20	0.20
	C2 Medicine costs	5.00 ± 1.5	7.76 ± 1.39	0.18	0.28
	C3 Incremental analysis	5.53 ± 1.58	7.41 ± 1.72	0.18	0.25
	Total	-	-	0.56	0.72
Innovation	D1 Medicine technology characteristics	7.88 ± 1.23	6.71 ± 1.27	0.18	0.15
	D2 Addressing unmet clinical needs	8.88 ± 1.23	5.88 ± 2.42	0.40	0.27
	D3 Industry information	8.29 ± 1.49	6.29 ± 1.99	0.12	0.09
	Total	-	-	0.70	0.51
Applicability	E1 Technical applicability	6.88 ± 1.41	7.76 ± 0.94	0.18	0.21
	E2 Usage applicability	6.94 ± 1.35	7.88 ± 1.08	0.29	0.33
	Total	-	-	0.48	0.54
Accessibility	F1 Accessibility	7.00 ± 1.19	8.35 ± 1.64	0.27	0.32
	F2 Affordability	5.82 ± 1.42	8.47 ± 0.98	0.23	0.34
	Total	-	-	0.50	0.66
Overall Score				7.40	7.19

SOC: standard of care; OMZ: omalizumab; ESc: each criterions score based on the evidence matrix; CS: comprehensive score. The values of ESc reported as mean ± standard deviation.

**Table 6:** Sensitivity analysis: varying assigned weights.

Domains	Sensitivity Analysis					
	1	2	3	4	5	6
Assigned weights (%)						
Safety	45.00	35.00	35.00	35.00	35.00	35.00
Effectiveness	25.00	35.00	30.00	30.00	30.00	30.00
Economics	10.00	10.00	15.00	10.00	10.00	10.00
Innovation	8.00	8.00	8.00	13.00	8.00	8.00
Applicability	5.00	5.00	5.00	5.00	10.00	5.00
Accessibility	7.00	7.00	7.00	7.00	7.00	12.00
CS						
OMZ + SOC	7.37	7.46	7.34	7.48	7.40	7.37
SOC	7.20	7.14	7.18	7.11	7.20	7.22

SOC: standard of care; OMZ: omalizumab; CS: comprehensive score. The numbers in the upper half of the table represented the assigned weights (%) of each domain. The numbers in the lower half of the table represented the strategies’ overall score calculated based on the upper weights combinations.

**Table 2:** Analysis of adverse event signals from 1678 omalizumab-related reports in the FAERS database.

Adverse Event	Frequency ^a^	PRR	χ^2^	ROR	95% CI	
Urticaria	223	11.60	2069.63	13.24	11.46	15.29
Allergic reaction	159	20.57	2708.34	22.63	19.09	26.83
Difficulty breathing	155	5.71	592.17	6.19	5.24	7.32
Headache	120	2.61	119.35	2.73	2.27	3.29
Lethargy	119	6.28	514.79	6.69	5.54	8.08
Cough	115	6.03	470.77	6.41	5.29	7.76
Wheezing	107	22.76	2007.95	24.25	19.76	29.76
Itching	103	5.34	354.89	5.63	4.60	6.88
Hypotension	97	23.70	1892.48	25.10	20.24	31.12
Weight gain	86	4.69	243.80	4.89	3.93	6.09

^a^ Frequency refers to the number of adverse event occurrences. ROR: reporting odds ratio; PRR: proportional reporting ratio.

## Data Availability

The data presented in this study are available upon request from the corresponding author. The data are not publicly available due to privacy.

## Supplementary Materials

The following supporting information can be downloaded at: https://www.mdpi.com/article/10.3390/healthcare13192385/s1, Figure S1: The PRISMA flow diagram of studies included in the SLR. SLR: systematic literature review; CNKI: China National Knowledge Infrastructure; VIP: China Science and Technology Journal Database; Table S1. The definition of the criteria; Table S2: The basic information of the seventeen experts; Table S3: The authority level of the seventeen experts.

## Abbreviations

The following abbreviations are used in this manuscript:

BA	Bronchial asthma
DALY	Disability adjusted life years
ICS	Inhaled corticosteroids
SOC	Standard of care
LABA	Long-acting B-beta2 agonists
HTA	Health technology assessments
MCDA	Multi-criteria decision analysis
RWD	Real-world data
AHP	Analytic hierarchy process
SLR	Systematic literature review
GETE	Global evaluation of asthma effectiveness
PAQLQ	Pediatric asthma quality of life questionnaire
C-ACT	Children’s asthma control test
ADR	Adverse drug reactions
CS	Comprehensive score
CPG	Clinical practice guidelines
IgE	Immunoglobulin E
GINA	Global Initiative for Asthma
Cr	Authority coefficients
PRR	Proportional reporting ratio
ROR	Reporting odds ratio
CSU	Chronic spontaneous urticaria
FAERS	FDA Adverse Event Reporting System
VAS	Visual analogue scale
