# Supplementary material for: Assessing the Value of Omalizumab for Pediatric Asthma in China: A Multicriteria Decision Analysis
Source: Healthcare (Basel). 2025 Sep 23;13(19):2385. doi: 10.3390/healthcare13192385 (PMC12523588; doi:10.3390/healthcare13192385)
Supplement: Supplementary file 1 [file healthcare-13-02385-s001.zip › healthcare-3808891-supplementary.pdf]

Supplementary Materials

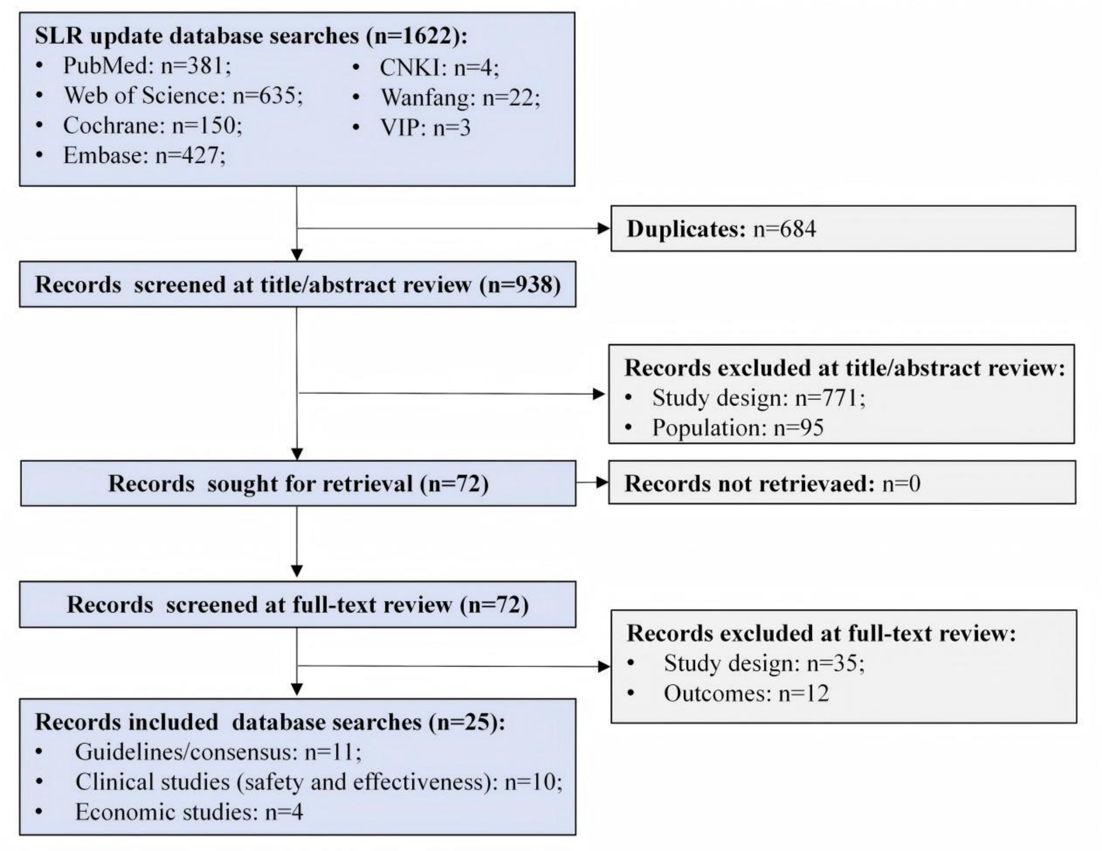

Figure S1. The flow diagram of studies included in the SLR. An initial search across multiple databases yielded 1,622 references. A total of 684 articles were excluded due to duplication. In addition, 866 articles were excluded based on title and abstract screening for reasons such as being review articles, lacking relevance to the topic, or focusing only on adult populations. The remaining 72 articles underwent full-text review. Of these, 35 were excluded due to inconsistent study designs and 12 were omitted for not reporting target outcomes. Ultimately, 25 studies were included in the analysis, comprising 11 guidelines/consensus statements, 10 clinical studies (evaluating safety and effectiveness), and 4 economic evaluations. SLR: systematic literature review; CNKI: China National Knowledge Infrastructure; VIP: China Science and Technology Journal Database.

Table S1. The definition of the criteria.

Domains	Criteria	Definition of the criteria
**A Safety**	A1 Pre-market safety	Pre-marketing genetic toxicity, reproductive toxicity, carcinogenicity, drug interactions, contraindications, drug overdoses, etc.
	A2 Adverse drug reactions/events	Post-marketing adverse reactions or adverse events.
	A3 Drug safety warnings	Drug safety warnings, such as market withdrawal, product recall, and package insert modifications, are issued by domestic and foreign drug regulatory authorities.
**B Effectiveness**	B1 Guideline recommendations	Recommendations from authoritative domestic and foreign guidelines or consensus.
	B2 Clinical efficacy	The degree of improvement in the patient's disease state following drug use. Primary or endpoint indicators are preferred, and alternative intermediate indicators may be used when primary indicators are not available.
**C Economics**	C1 Economic reports	Economic reports published by authoritative domestic and foreign institutions.
	C2 Drug costs	The direct, measurable price of a drug, focusing on the financial expenditure required to purchase and administer the drug.
	C3 Incremental analysis	Basic health economics methods can be used to analyze drug costs and benefits and measure their economic value, including cost-utility analysis, cost-effectiveness analysis, cost-benefit analysis, minimum cost analysis, etc.
**D Innovation**	D1 Drug technology characteristics	Whether the drug has significant technical advantages over other similar substitutable drugs, such as formulation stability, bioavailability, pharmacokinetic characteristics, efficacy, dosage form, taste, administration method, specifications, etc.
	D2 Addressing unmet clinical needs	Whether the drug can meet the health needs that other substitutable drugs fail to meet.
	D3 Industry information	Whether domestic or international patents are obtained for the drug's structure or formulation.
**E Applicability**	E1 Technical applicability	Completeness of labeling, the indication of dosage for children, suitability of dosage form and taste, storage conditions, special devices, and the need for monitoring or follow-up after administration.
	E2 Usage applicability	(1) rational use: whether the drug is targeted for indications, whether there is sufficient reason for off-label use, whether combination therapy is reasonable, and whether contraindications are accurately excluded;

		(2) Medication applicability: whether the drug dose meets requirements, whether the route of administration, administration time and intervals, and treatment duration are appropriate, and whether it complies with guidelines, etc.
**F Accessibility**	F1 Accessibility	The potential opportunities for children to access drugs include drug policies, availability in medical institutions and drug retail, drug shortage status, number of distribution companies, distribution channels, and capabilities.
	F2 Affordability	The affordability is assessed by comparing annual per capita drug treatment costs per year to the annual per capita disposable income of urban and rural families.

Table S2. The basic information of the seventeen experts.

Characteristics	Classification	Number of experts	Percentage of experts (%)
**Professional**	Clinical	7	41.18
	Pharmacy	6	35.29
	Methodology	4	23.53
**Age (Years)**	30-40	9	52.94
	41-50	4	23.53
	51-60	4	23.53
**Work Experience**	Less than 10 years	4	23.53
	10-20 years	8	47.06
	20-30 years	3	17.65
	More than 30 years	2	11.76
**Title**	Senior	7	41.18
	Associate Senior	9	52.94
	Middle	1	5.88
**Regional Distribution**	Beijing	10	58.83
	Tianjin	1	5.88
	Shandong	1	5.88
	Henan	1	5.88
	Anhui	1	5.88
	Jiangsu	2	11.76
	Shanxi	1	5.88

Table S3. The authority level of the seventeen experts.

Experts ID	Safety	Effectiveness	Economics	Innovation	Applicability	Accessibility
**1**	0.85	0.85	0.60	0.70	0.85	0.80
**2**	0.90	0.90	0.85	0.65	0.65	0.85
**3**	1.00	1.00	1.00	0.95	0.95	0.90
**4**	1.00	1.00	0.65	0.70	0.90	0.90
**5**	1.00	1.00	0.70	0.75	0.85	0.95
**6**	1.00	1.00	0.95	0.95	0.95	1.00
**7**	1.00	0.95	1.00	0.95	0.95	1.00
**8**	1.00	1.00	0.90	0.65	0.80	0.90
**9**	0.95	1.00	0.70	0.80	0.85	0.70
**10**	0.90	1.00	0.90	1.00	0.85	1.00
**11**	1.00	1.00	0.95	0.90	0.85	0.85
**12**	0.90	0.90	0.90	0.90	0.90	0.90
**13**	1.00	1.00	0.95	0.95	0.90	0.90
**14**	0.75	0.75	0.95	0.70	0.70	0.70
**15**	0.80	0.80	0.70	0.65	0.65	0.65
**16**	1.00	0.95	0.95	0.80	0.70	0.80
**17**	1.00	0.95	1.00	0.95	0.95	1.00
Mean	0.94	0.94	0.86	0.82	0.84	0.87
